# Subepithelial corneal fibrosis partially due to epithelial-mesenchymal transition of ocular surface epithelium

**Published:** 2010-12-15

**Authors:** Motoko Kawashima, Tetsuya Kawakita, Kazunari Higa, Yoshiyuki Satake, Masahiro Omoto, Kazuo Tsubota, Shigeto Shimmura, Jun Shimazaki

**Affiliations:** 1 Department of Ophthalmology, Keio University School of Medicine, Tokyo, Japan; 2 Department of Ophthalmology, Tokyo Dental College, Chiba, Japan

## Abstract

**Purpose:**

To determine whether epithelial-mesenchymal transition is involved in the development of corneal subepithelial fibrosis (pannus).

**Methods:**

Frozen samples of pannus tissue removed from human corneas with a diagnosis of total limbal stem cell deficiency were characterized by immunostaining for both epithelial and mesenchymal markers. We selected transformation-related protein 63 (p63) and pancytokeratin as epithelial markers and vimentin and α-smooth muscle actin (α-SMA) as mesenchymal markers. Immunostaining for β-catenin and E-cadherin was performed to determine wingless-Int (Wnt)-pathway activation. RT–PCR analysis was also performed on epithelial tissue obtained from pannus samples after dispase digestion.

**Results:**

Immunohistochemistry revealed strong nuclear expression of p63 and weak intercellular expression of E-cadherin in epithelial basal cells of pannus tissue. Furthermore, translocation of β-catenin from intercellular junctions to the nucleus and cytoplasm was also observed. Double-positive cells for both p63 and α-SMA were observed in the subepithelial stroma of pannus tissue, which was supported by RT–PCR and cytospin analysis.

**Conclusions:**

Epithelial-mesenchymal transition may be partially involved in the development of subepithelial corneal fibrosis due to total limbal stem cell deficiency.

## Introduction

Fibrosis is a common pathologic event observed in various organs of the body, including lung and kidney. Recent studies have demonstrated that the cellular origins of fibroblasts during disease are found not only in remnants of embryonic development, but also in tissue-specific epithelial cells and circulating cellular pools [1-5]. In particular, there is the potential for epithelial cells to undergo a change in phenotype, differentiating into fibroblastic cells in response to morphogenic pressure from injured tissue in what is known as epithelial–mesenchymal transition (EMT). Recently, it has been reported that EMT occurred in a limbal location in rabbit corneal explant [6], and was also involved in the fibrotic process in mouse corneal wound healing after alkali burn, mouse traumatic cataract [7], mouse retinal detachment [8], and human pterygium [9]. Abnormal subepithelial fibrosis and epithelial keratinization, in particular, can cause vision-threatening diseases such as severe ocular surface fibrosis due to total limbal stem cell deficiency [10,11]. Histopathologically, corneas with total limbal stem cell deficiency are characterized by conjunctival ingrowth (conjunctivalization), vascularization and chronic inflammation. Such phenomena may originate from severe inflammation due to corneal burn, Stevens-Johnson syndrome, or ocular cicatricial pemphigoid. These diseases completely destroy limbal epithelial stem cells, their surrounding environment, or a combination of both.

Generally, the mechanism of corneal subepithelial fibrosis is initiated by corneal stromal fibroblast activation due to inflammatory cytokines [12,13]. This activation may then lead to transdifferentiation into α-smooth muscle actin (α-SMA)-positive myofibroblasts [14]. Contraction of the stress fibers in myofibroblasts subsequently results in the development of fibrosis [13-15]. We hypothesized that abnormal ocular surface epithelial cells might also contribute to the fibrotic changes observed in limbal stem cell deficiency. To test this hypothesis, we analyzed human corneal subepithelial fibrosis samples with limbal stem cell deficiency.

## Methods

### Patients

**Figure 1:**
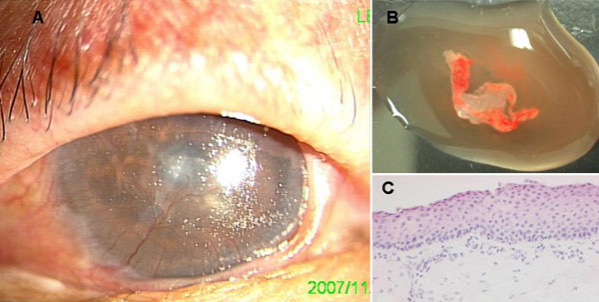
Histopathology of pannus. Clinical appearance of patient (case 7) showed thin pannus covering whole cornea (**A**). Removed pannus in case 7 (**B**). Representative pannus photo of hematoxylin-eosin staining (**C**) showing irregular epithelial basal layer and hyper-epithelialization associated with increased subepithelial fibroblasts.

Ten patients with a clinical diagnosis of total limbal stem cell deficiency confirmed by impression cytology were included in the study. In some of the patients, an obvious clinical diagnosis of total limbal stem cell deficiency obviated the need for cytological confirmation (Figure 1A). The etiologies included chemical burn (n=3), Stevens-Johnson syndrome (n=4), ocular cicatricial pemphigoid (n=1), pseudo-ocular cicatricial pemphigoid (n=1), and aniridia (n=1; Table 1). None of the patients had undergone surgical procedures for ophthalmological disorders before receiving ocular surface reconstruction at our facility. With prior written informed consent, clinical samples of pannus were obtained from these patients intraoperatively.

**Table 1:** Demographic data.

**Case**	**Age**	**Sex**	**Original disease**	**Examination**
1	34	M	Chemical burn	IHC
2	33	F	Chemical burn	IHC
3	10	M	AB	IHC
4	80	F	SJS	IHC
5	42	F	SJS	IHC
6	47	M	SJS	IHC
7	69	M	OCP	IHC
8	82	F	pOCP	IHC
9	62	F	aniridia	RT–PCR
10	57	M	SJS	RT–PCR

AB=alkali burn, SJS=Stevens-Johnson syndrome, OCP=ocular cicatricial pemphigoid, OCP=pseudo-OCP, IHC=immunohistochemistry.

**Figure 2:**
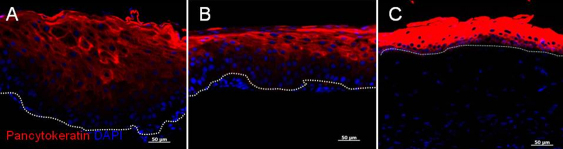
Immunostaining of pannus tissue against pancytokeratin (CK22). Epithelium of pannus in all cases stained positive for pancytokeratin (red), particularly in superior hyperepithelial layers. However, basal cells showed weak staining (**A** and **B**). **C**: pancytokeratin staining in normal cornea as a control. The scale bar indicates 50 μm.

The pannus specimens (Figure 2B) were immediately embedded and frozen for sectioning and maintained at −80 °C for immunostaining. After dispase digestion, Reverse Transcription-Polymerase Chain Reaction (RT–PCR) was used to analyze two samples of obtained epithelia from pannus. For cytospin, separately prepared pannus samples were digested with dispaseII for 1 h at 37 °C and Trypsin-EDTA at 37 °C for 5 min. Cytospin-slides were stored at −80 °C for immunostaining.

Normal human corneas were obtained from Northwest Lions Eyebank (Seattle, WA) as a control. All research protocols were approved by the appropriate ethics committees of Tokyo Dental College and all procedures performed in accordance with the tenets of the Declaration of Helsinki.

### Histology and immunofluorescent staining

A normal section comprising cornea and limbus was used as the control. Multiple 5-μm-thick frozen sections were prepared on gelatin-coated slides, fixed with 2% paraformaldehyde for 5 min and reacted with anti–p63 (Santa Cruz Biotechnology, Inc. Santa Cruz,CA), anti-β-catenin (Santa Cruz, CA), anti-E-cadherin (Santa Cruz CA), anti-pan cytokeratin (Abcam, Cambridge, UK), anti-α-SMA (Abcam), anti-vimentin (Neomarkers, Fremont, CA) and anti-collagen 4 (Southern Biotec, Birmingham, AL) antibodies. Sections were then treated with Cy3- or FITC-, Alexa Fluor 488-conjugated secondary antibodies (Chemicon International, Temecula, CA). Cell nuclei were counterstained with DAPI (1 µg/ml; Dojindo Laboratories, Tokyo, Japan). For histological examination, hematoxylin eosin staining was performed according to the standard procedure. Double immunostaining for p63 and α-SMA was performed in the cytospin-slides.

### Reverse transcription–polymerase chain reaction

After digestion by dispase, total RNA was extracted from epithelial cells using a commercial kit (RNeasy; Qiagen, Hilden, Germany), followed by cDNA synthesis (RevaTra Ace-- kit; Toyobo Co. Ltd., Osaka, Japan). PCR was performed (GeneAmp 9700; Applied Biosystems, Foster City, CA) with the Advantage 2 PCR Enzyme System (Clontech, Takara Bio Inc., Shiga, Japan) as follows: 95 °C for 5 min, 2 cycles of 95 °C for 90 s, 57 °C for 30 s, and 72 °C for 30 s, and 28 cycles of 95 °C for 30 s, 57 °C for 30 s, and 72 °C for 30 s. Sequences for primers are shown in Table 2. PCR products were analyzed by gel electrophoresis.

**Table 2:** PCR primer sequences.

** **	**Sequence**		** **	** **
**Name**	**Forward**	**Reverse**	**Product size (bp)**	**Melting temperature**
α-SMA	ACTGGGACGACATGGAAAAG	CATCTCCAGAGTCCAGCACG	241	60
matrix metalloproteinase (MMP)-2	AGATCTTCTTCTTCAAGGACCGGTT	GGCTGGTCAGTGGCTTGGGGTA	225	65
MMP9	GCGGAGATTGGGAACCAGCTGTA	GACGCGCCTGTGTACACCCACA	208	64
E-cadherin	TCGACACCCGATTCAAAGTGG	TTCCAGAAACGGAGGCCTGAT	194	58
beta-Catenin	GCTGATTTGATGGAGTTGGA	GCTACTTGTTCTTGAGTGAA	226	54
Snail-1	TATGCTGCCTTCCCAGGCTTG	ATGTGCATCTTGAGGGCACCC	145	61
GAPDH	ACCACAGTCCATGCCATCAC	TCCACCACCCTGTTGCTGTA	452	58

## Results

### Histopathology and immunohistochemistry of pannus

**Figure 3:**
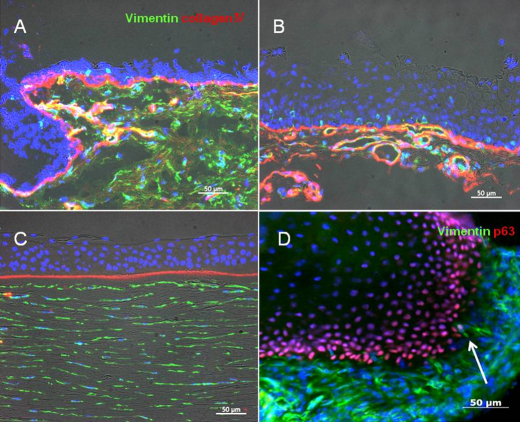
Immnostaining of pannus tissue against vimentin and collagen IV. Compared to control (**C**, normal cornea), collagen IV expression (red) was irregular in pannus, and was also found in vessel walls (**A** and **B**). Vimentin(+) (green) stromal cells were uniformly observed in normal central cornea, but supra- and sub-epithelial activated vimentin(+) cells were observed in pannus. Cells double-positive for vimentin and p63 were observed (**D**, indicated by arrow). The scale bar indicates 50 μm.

**Figure 4:**
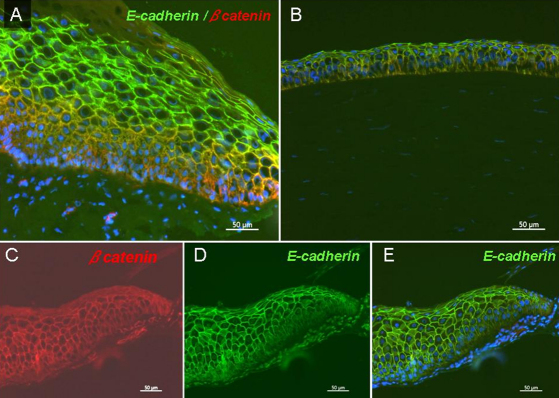
Immunohistochemistry of E-cadherin and β-catenin. Epithelial basal cells in pannus showed weak staining for E-cadherin (green) and translocation of β-catenin (red) from intercellular junctions to cytoplasm. **A**: Pannus with double-staining for E-cadherin and β-catenin. **B**: Normal cornea. **C**: βcatenin, **D**: E-cadherin, **E**: E-cadherin and DAPI, in pannus epithelium was shown. **A** was a combination of **C**, **D**, and **E**. The scale bar indicates 50 μm.

**Figure 5:**
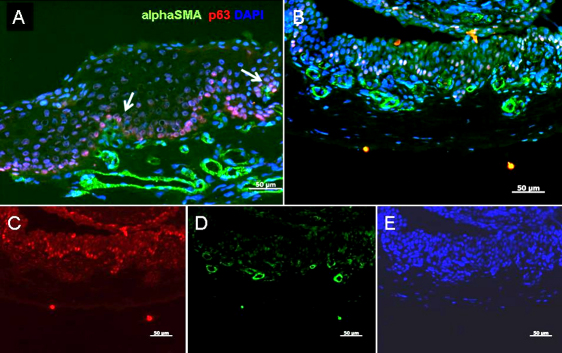
Immunostaining of pannus tissue against p63 and α-SMA. Nuclear p63 (red) and cytoplasmic α- SMA (green) double-positive cells were observed in subepithelial location in pannus tissue (**A** and **B**). **B** was syntheses photo of p63 (**C**), α- SMA (**D**), and DAPI (**E**). Scale bar indicates 50 μm.

**Figure 6:**
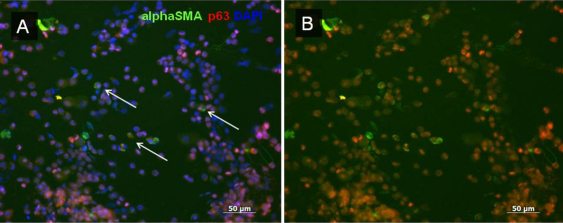
Immunostaining of cytospin of pannus tissue. Double-immunostaining for p63 (red) and α-SMA (green) was observed in individual cells (indicated by arrows) in cytospin of pannus (**A**, with DAPI staining; **B**, without DAPI staining).  Scale bar indicates 50 μm.

We investigated whether EMT had occurred in pannus tissue by immunohistochemical analysis. For phenotypic analysis, vimentin and α-SMA were selected as mesenchymal markers and pan-cytokeratin (K) and p63 as epithelial markers. Nuclear and cytoplasmic translocation of β-catenin expression was also determined as a marker for EMT. Full-thickness epithelium in the pannus and normal corneal epithelium tested positive for pancytokeratin in all cases, although the strength of staining differed. Basal cells showed very weak staining for pancytokeratin (Figure 2). Compared to the control, the basal membrane was irregular and subepithelial vascularization was noted in pannus (Figure 3). In normal cornea, collagen type 4 showed a regular pattern of expression in the basement membrane layer, as indicated by the red line in Figure 3C. However, in pannus tissue and the vessel walls, expression of collagen type 4 showed an increase or was irregular. Vimentin(+) stromal cells were uniformly observed in normal central cornea, whereas supra- and sub-epithelial-activated vimentin(+) cells were observed in pannus tissue (Figure 3). Furthermore, double-positive cells for both epithelial marker nuclear p63 and mesenchymal marker vimentin were observed in pannus tissue (Figure 3D). As shown in Figure 4, intact corneal epithelium was positive for membrane E-cadherin and β-catenin. Epithelial basal cells in pannus tissue showed weak expression of E-cadherin and translocation of β-catenin from intercellular junctions to the cytoplasm (Figure 4), indicating at least partial dissolution of cell-cell junctions. No expression of α- SMA was observed in intact normal cornea by immunohistochemistry (data not shown). Double-positive cells for both epithelial marker nuclear p63 and mesenchymal marker cytoplasmic α-SMA were observed in subepithelial locations in pannus tissue (Figure 5). Furthermore, cytospin of pannus epithelium revealed cells with positive immunostaining for both nuclear p63 and cytopkasmic α-SMA, which supported the findings in the tissue sections (Figure 6).

### RT–PCR results

**Figure 7:**
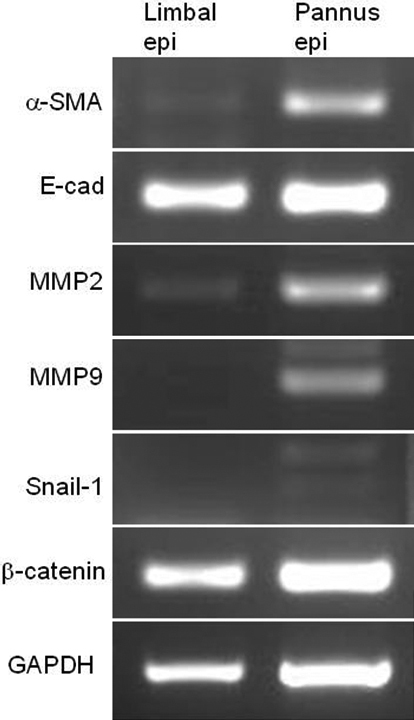
RT-PCR of pannus epithelium. *α-SMA*, *MMP-2*, and *MMP-9* were all expressed in pannus epithelium, but not in limbal epithelium.  Weak *Snail-1* expression was observed in pannus epithelium. E-cadherin and β-catenin were expressed in both  limbal epithelial cells and pannus.

Weak expression of *α-SMA* and *MMP-2* and no expression of *MMP-9* were observed in normal limbal epithelial cells. On the other hand, motility-associated proteins *α-SMA*, *MMP-2*, and *MMP-9* were all expressed in samples of pannus tissue (representative data, Figure 7), which supported the immunostaining data. Weak *Snail-1* expression was also observed in pannus tissue. E-cadherin and β-catenin were expressed in both normal limbal epithelial cells and pannus.

## Discussion

The results of this study indicate that EMT may be involved in the development of corneal pannus due to limbal stem cell deficiency. Kawakita et al. [6] previously reported that rabbit corneal limbal epithelial cells underwent EMT and invaded underlying stroma after exposure to air in a rabbit limbal explant model. This suggests that putative corneal epithelial progenitor cells in the basal limbal layer can differentiate into mesenchymal cells, which would agree with the histologic findings on fibrosis in this study. Therefore, it is reasonable to hypothesize that EMT in altered limbal epithelial cells is involved in the pathogenesis of pannus.

Fibrotic diseases of the ocular surface, including corneal and conjunctival scar, have been recognized as having a mechanism quite similar to that of fibrotic disorders in other tissues of the human body. Inflammation is thought to trigger and facilitate the progression of fibrosis. Subsequently, epithelial cells are stimulated and injured without appropriate repair, resulting in activation of underlying fibroblasts. These fibroblasts derive from bone marrow, but may also arise as a result of EMT in cells at injury sites [16]. This process of fibrotic tissue transformation is characterized by an increase in abnormal extracellular matrix, including collagen fibers. Such an excess accumulation of extracellular matrix and the appearance of α-SMA-positive myofibroblasts and inflammatory cells were observed in all the pannus samples in this study. Several studies have reported α- SMA as a marker for EMT during fibrosis [17-19]. No expression of α-SMA was observed in intact cornea. Although it is not clear whether they were vessel cell-derived or not in this study, double-positive cells for both nuclear p63 and cytoplasmic α-SMA were observed in subepithelial locations in pannus tissue (Figure 5). Moreover, cytospin revealed both p63 and α-SMA in pannus tissue-derived cells (Figure 6). Taken together, these results suggest that EMT occurs in pannus tissue.

The process of EMT requires alterations in morphology, cellular architecture, adhesion and migration capacity. Commonly used molecular markers for EMT include increased expression of N-cadherin and vimentin, nuclear localization of β-catenin and increased production of transcription factors such as Snail1 (Snail) and Snail2 (Slug), which inhibit E-cadherin production [1-5,20-24]. Phenotypic markers for EMT include an increased capacity for migration and 3-dimensional invasion, as well as resistance to anoikis/apoptosis [1-5]. MMP-2 and −9 have been shown to be important in both normal corneal wound healing and EMT-like changes in tumor formation [25,26]. In this study, EMT was evidenced by morphological alteration and expression of the fibroblast phenotypic marker vimentin, concomitant with downregulation of epithelial phenotype marker E-cadherin. Expression of *Snail 1*, *MMP-2*, and *MMP-9* RNA was also upregulated (Figure 7). Epithelial basal cells in pannus tissue showed weak expression of E-cadherin and translocation of β-catenin from intercellular junctions to the cytoplasm in the immunohistological examination. This agreed with the results of earlier studies showing activation of the Wnt signaling pathway, a major signal transduction pathway of EMT [2,9,27,28]. However, RT–PCR levels of E-cadherin and β-catenin showed no change between pannus epithelial cells and control limbus cells in this study, which may have been due to hyperkeratinization.

In this context, MT1-MMP plays an important role by activating other MMPs such as proMMP-2 at the cell surface [29,30]. Activation of proMMP-2 by MT1-MMP is considered to be a pivotal step in tumor cell invasion, as activated MMP-2 degrades collagen type IV and laminin, the major components of basement membranes [31,32]. Increase in corneal epithelial MMP-2 expression in pannus tissue may be a step in the EMT process.

In this study, we demonstrated that the Wnt/β-catenin pathway was activated in basal epithelial cells in human pannus samples. Proteases such as MMP2 might play a major role in the migration/invasion process of EMT. The number of α-SMA-positive, activated (myo) fibroblasts increased in pannus fibrosis. However, their origin remains to be elucidated in further study.
